# Presentations of tetramethylammonium hydroxide dermal exposure and the valuable potential of diphoterine solution in decontamination: a retrospective observational study

**DOI:** 10.1186/s40360-020-00465-8

**Published:** 2020-11-30

**Authors:** Chih-Kang Huang, Alan H. Hall, Ming-Ling Wu, Chen-Chang Yang, Dong-Zong Hung, Yan-Chiao Mao, Jou-Fang Deng

**Affiliations:** 1 Department of Emergency Medicine, Tri-Service General Hospital, National Defense Medical Center, Taipei, Taiwan; 2 Toxicology Consulting and Medical Translating Services, Azle and Springtown, TX USA; 3 Institute of Environmental and Occupational Health Sciences, School of Medicine, National Yang-Ming University, Taipei, Taiwan; 4 Division of Clinical Toxicology and Occupational Medicine, Department of Medicine, Taipei Veterans General Hospital, Taipei, Taiwan; 5 Department of Toxicology, China Medical University Hospital, Taichung, Taiwan; 6 Division of Clinical Toxicology, Department of Emergency Medicine, Taichung Veterans General Hospital, Taichung, Taiwan

**Keywords:** Tetramethylammonium hydroxide, TMAH, Diphoterine® solution, Dermal exposure, Dermal decontamination, Skin decontamination

## Abstract

**Background:**

Tetramethylammonium hydroxide (TMAH) is a quaternary ammonium compound that is both a base corrosive and a cholinergic agonist, and it is widely used in the photoelectric and semiconductor industries. It causes corrosive skin injuries and systemic cholinergic toxicity with death primarily resulting from respiratory failure without efficacious early decontamination.

**Methods:**

A retrospective observational study was performed of all cases of TMAH exposure reported to the Taiwan Poison Control Center between July 2010 and October 2017. Retrieved medical records were independently reviewed by two trained clinical toxicologists.

**Results:**

Despite immediate (< 5 min) skin decontamination with copious amounts of tap water, one patient exposed to 25% TMAH involving ≥5% of total body surface area (TBSA) developed significant systemic toxicity. Patients exposed to 25% TMAH involving ≤1% TBSA developed first-degree chemical skin injuries but no systemic toxicity. Among patients exposed to lower concentrations (≤2.38%) of TMAH, the majority only experienced first-degree chemical skin injuries without systemic signs. Patients exposed to 0.5% TMAH involving nearly their entire TBSA developed no chemical skin injuries or systemic toxicity. All patients who had only first-degree chemical skin injuries did not develop systemic toxicity after exposure to either 2.38% or 25% TMAH.

**Conclusions:**

TMAH acts as an alkaline corrosive and cholinergic agonist. Systemic signs attributable to TMA^+^ can rapidly lead to respiratory failure and death after dermal exposure. We have demonstrated that an amphoteric solution may be efficacious for skin decontamination on-site immediately to prevent or ameliorate such toxicity. This practice especially carries a valuable potential in managing victims (patients) who have been exposed to those chemicals with immediate life-threatening toxicity (e.g. TMAH), suggesting that its early utilization deserves further study.

## Background

Tetramethylammonium ion (TMA^+^, C_4_H_13_N^+^), a simple quaternary ammonium compound, was first isolated and identified from a sea anemone in 1923 [1]. Its hydroxide salt, tetramethylammonium hydroxide (TMAH), is commonly used in the semiconductor and photoelectric industries as a developer and as an agent for silicon anisotropic etching. In the US, TMAH is listed as a high production volume chemical. In 2002, the US production of TMAH totaled 10–50 million pounds [2, 3]. Meanwhile, many semiconductor facilities utilizing TMAH are located in China, Japan, South Korea, and Taiwan [4].

TMAH is recognized as an alkaline corrosive and a cholinergic agonist that can cause both chemical skin injury and systemic toxicity, as indicated by acute respiratory failure in a rat model [5]. Fatalities have been reported after dermal exposure to TMAH [6–10]. Clinically, the features of systemic TMAH intoxication include generalized weakness, excessive salivation, and dyspnea, which develop within 10–15 min following dermal exposure [6, 7]. Out-of-hospital cardiac arrest has been reported even when patients were immediately decontaminated with copious amounts of tap water [6, 7, 10].

No antidote for TMAH poisoning is currently available. Efficacious early decontamination can play an important role in preventing or ameliorating TMAH-related systemic toxicity. A worker (Case 1 in Table 1) who was exposed to 25% TMAH in an industrial accident developed first-to-second-degree chemical skin injuries over 5% TBSA. He reached the automatic tap water flushing system (40 m away) approximately 5 min after the exposure. Despite flushing with a copious amount of tap water, the worker developed systemic manifestations of dyspnea, drowsiness, palpitations, weakness, and bradycardia. Within a few minutes, a co-worker brought Diphoterine® solution to assist with further decontamination. Approximately 5 min later while using the 4th and 5th 100-mL Diphoterine® solution spray containers, the patient’s consciousness cleared and he expressed pain in the exposed areas. Thereafter, he was admitted to a local medical center and was discharged 3 days later without any sequela.

**Table 1:** Summary of 29 patients with dermal tetramethylammonium hydroxide (TMAH) exposure in Taiwan

Case No	Age/sex	Concentration of TMAH (%)	Exposed BSA	Elapsed time to decontamination	Decontamination time	Diphoterine	Clinical manifestations	Laboratory abnormalities	Treatment/outcome
1	34/M	25%	5% BSA	5 min	> 15 min	Used	First to second degree chemical burn, dyspnea, drowsiness, bradycardia	Leukocytosis, hypokalemia	Supportive, intensive care /survived
2	29/M	25%	2% BSA	N/A	15 min	None	Second-degree chemical burn	All normal	Supportive, intensive care /survived
3	22/M	25%	1% BSA	< 1 min	N/A	Used	First-degree chemical burn	All normal	Supportive/survived
4	27/M	25%	< 1%BSA	< 1 min	N/A	Used	First-degree chemical burn	All normal	Supportive/survived
5	46/M	25%	< 1% BSA	N/A	N/A	None	First-degree chemical burn	N/A	Supportive/survived
6	48/M	25%	1% BSA	N/A	N/A	None	First-degree chemical burn	N/A	Supportive/survived
7	23/M	20% diluted	1% BSA	< 1 min	N/A	Used	First-degree chemical burn	All normal	Supportive/survived
8	25/M	2.38%	< 1% BSA	N/A	N/A	None	First-degree chemical burn	N/A	Supportive/survived
9	25/F	2.38%	< 1% BSA	N/A	N/A	Used	First-degree chemical burn	N/A	Supportive/survived
10	36/M	2.38%	< 1% BSA	N/A	N/A	None	First-degree chemical burn	N/A	Supportive/survived
11	28/M	2.38%	< 1% BSA	N/A	N/A	None	First-degree chemical burn	N/A	Supportive/survived
12	23/F	2.38%	< 1% BSA	N/A	N/A	None	None	None	Supportive/survived
13	35/M	2.38%	1% BSA	N/A	N/A	None	First-degree chemical burn	None	Supportive/survived
14	29/M	2.38%	< 1% BSA	< 5 min	30 min	None	None	None	Supportive/survived
15	37/M	2.38%	< 1% BSA	< 5 min	30 min	None	None	None	Supportive/survived
16	33/M	2.38%	< 1% BSA	N/A	N/A	None	First-degree chemical burn	None	Supportive/survived
17	34/M	2.38%	2% BSA	N/A	N/A	None	First-degree chemical burn	None	Supportive/survived
18	36/M	2.36%	< 2% BSA	N/A	N/A	None	None	All normal	Supportive/survived
19	43/M	2.38%	N/A (Right arm)	N/A	N/A	None	First-degree chemical burn	N/A	Supportive/survived
20	23/M	0.50%	Nearly entire body	30 min	20 min	None	None	All normal	Supportive/survived
21	26/M	0.50%	Nearly entire body	30 min	20 min	None	None	Leukocytosis	Supportive/survived
22	27/M	N/A	< 1% BSA	N/A	N/A	None	First-degree chemical burn	N/A	Supportive/survived
23	22/M	N/A	1% BSA	N/A	N/A	None	First-degree chemical burn	N/A	Supportive/survived
24	34/M	N/A	< 1% BSA	N/A	20 min	None	None	All normal	Supportive/survived
25	39/M	N/A	< 1% BSA	N/A	15 min	None	First-degree chemical burn	All normal	Supportive/survived
26	30/M	3%	N/A (Bil. forearm)	N/A	N/A	None	None	All normal	Supportive/survived
27	27/F	N/A	< 1% BSA	40 min	30 min	None	First-degree chemical burn	None	Supportive/survived
28	25/F	N/A	< 1%BSA	N/A	N/A	None	First-degree chemical burn	All normal	Supportive/survived
29	22/F	1–3%	< 1%BSA	None	None	None	First-degree chemical burn	N/A	Supportive/survived

In this retrospective observational study, we analyzed the relationships between systemic toxicity and the extent of TMAH exposure, and between systemic toxicity and the degree of chemical burn. In addition, we discussed the valuable potential of Diphoterine solution to be used in decontamination of TMAH splashes.

## Methods

### Study methods

A retrospective observational study was performed by collecting data reported by telephone to the Taiwan Poison Control Center (PCC-Taiwan). All relevant inquiries made to PCC-Taiwan from July 2010 to October 2017 were identified by searching the computer database of PCC-Taiwan using the terms TMA and TMAH in both English and Mandarin. The identified retrieved data were then manually reviewed by two trained clinical toxicologists independently.

### Dermal exposure to TMAH

#### Inclusion criteria

The dermal exposure of TMAH was confirmed by the exposure history and identification of the agent by the physicians in charge.

#### Exclusion criteria

Patients with no TMAH exposure and other medical condition inquiries were excluded.

### Data Extracted from PCC-Taiwan records

Age, gender, the TMAH concentration, the total body surface area (TBSA) exposed to TMAH, the elapsed time to decontamination, the decontamination time, the usage of Diphoterine, clinical manifestations, laboratory abnormalities, treatment, and outcome were abstracted onto a standardized form.

### Form development

The form was developed as follows. Prior to 2008, three TMAH-exposed workers who died before reaching the emergency department (within 30 min post-exposure) were identified. PCC-Taiwan was then contacted to obtain information regarding the management of such cases. Toxicokinetic and toxicodynamic data for TMAH were not available. This motivated the staff of PCC-Taiwan to analyze cases of TMAH exposure, resulting in the publication of a case series in 2010 [7] and the development of a modified consensus form featuring the same format shown in Table 1.

## Results

Over a 7-year period, 29 cases of dermal TMAH exposure were reported to PCC-Taiwan. The demographic data are highlighted in Table 2. The mean age was 31 years and 24 patients (82.9%) were male. The majority of exposed concentrations of TMAH were 2.38% (11 patients, 37.9%) and 25% (6 patients, 20.7%). The majority of exposed TBSA were < 1% (16 patients, 55.2%) and 1% (5 patients, 17.2%). Three patients (10.3%) spent > 30 min before portable decontamination and 6 patients (20.6%) spent < 5 min before decontamination. Three patients (10.3%) received decontamination for 30 min and 6 patients (20.6%) received decontamination for 15–20 min. Five patients (17.2%) used Diphoterine®. The clinical characteristics are shown in Table 3. Nineteen patients (65.5%) had first-degree chemical burns and 2 patients (6.9%) had second-degree chemical burns. One patient (3.4%) developed dyspnea, drowsiness, and bradycardia. The severity of poisoning and outcomes are outlined in Table 4. One (3.4%), 1 (3.4%), and 27 patients (93.1%) presented with severe, moderate, and minor intoxication, respectively. Two patients (6.9%) were admitted to the intensive care unit and 27 patients (93.1%) were medically discharged.

**Table 2:** Patient characteristics

	Number of cases,
	*N* = 29 (%)
Sex	
Male	24 (82.8%)
Female	5 (17.2%)
Age (years)	
21–30	17 (58.6%)
31–40	9 (31%)
> 40	3 (10.3%)
Concentration of TMAH (%)	
25%	6 (20.7%)
20% diluted	1 (3.4%)
3%	1 (3.4%)
2.38%	11 (37.9%)
2.36%	1 (3.4%)
1–3%	1 (3.4%)
0.50%	2 (6.9%)
N/A	6 (20.7%)
Exposed BSA(%)	
> 5%	2 (6.9%)
5%	1 (3.4%)
2%	2 (6.9%)
2 ~ 1%	1 (3.4%)
1%	5 (17.2%)
< 1%	16 (55.2%)
N/A	2 (6.9%)
Elapsed time to decontamination	
40 min	1 (3.4%)
30 min	2 (6.9%)
5 min	1 (3.4%)
5 min–1 min	2 (6.9%)
< 1 min	3 (10.3%)
None	1 (3.4%)
N/A	19 (65.5%)
Decontamination time	
30 min	3 (10.3%)
20 min	3 (10.3%)
20 min–15 min	1 (3.4%)
15 min	2 (6.9%)
None	1 (3.4%)
N/A	19 (65.5%)
Diphoterine	
Used	5 (17.2%)
None	24 (82.8%)

**Table 3:** Clinical characteristics

	Number of cases
	N = 29 (%)
First-degree chemical burn	19 (65.5%)
Second-degree chemical burn	2 (6.9%)
Dyspnea	1 (3.4%)
Drowsy	1 (3.4%)
Bradycardia	1 (3.4%)
None	8 (27.6%)

**Table 4:** Severity of poisoning and outcome

	Number of cases
	N = 29 (%)
Severity of poisoning	
Minor	27 (93.1%)
Moderate	1 (3.4%)
Severe	1 (3.4%)
Fatal	0 (0%)
Outcome	
Medically discharged home	27 (93.1%)
Admission to intensive care unit	2 (6.9%)
Admission to ordinary ward	0 (0%)

One worker who was exposed to 25% TMAH developed signs including first-to-second-degree chemical skin injuries and systemic manifestations of dyspnea, drowsiness, palpitations, and bradycardia (Case 1 in Table 1). Another worker exposed to 25% TMAH involving 2% TBSA developed second-degree chemical burns without systemic signs (Case 2 in Table 1). Four workers exposed to 25% TMAH involving ≤1% TBSA developed only first-degree chemical skin injuries without systemic signs (Cases 3–6 in Table 1). Patient exposed to 25% TMAH involving ≥5% TBSA developed significant systemic toxicity, whereas those exposed to 25% TMAH involving ≤1% TBSA developed only localized injuries.

In patients exposed to lower concentrations (≤2.38%) of TMAH, the majority had only first-degree chemical skin injuries without systemic signs (Cases 8–11, 13, 16–17, 19 in Table 1). Two workers exposed to 0.5% TMAH involving nearly their entire TBSA who were decontaminated with tap water approximately 30 min after exposure developed no chemical skin injuries or systemic toxicity (Cases 20–21 in Table 1).

All patients who had only first-degree chemical skin injuries did not develop systemic toxicity after exposure to either 2.38 or 25% TMAH in this retrospective observational study (Cases 3–11, 13, 16–17, 19, 22–23, 25, 27–29 in Table 1).

## Discussion

While an intact skin can protect us from penetration of foreign chemicals into our body, it is not a perfect barrier and skin penetration by various chemical substances can cause dermal injury and sometimes serious systemic poisoning. However, once the skin is injured or corroded by an acid or alkali, it will allow chemical compound to enter the body more easily, and consequently may result in significant toxicity. Upon a chemical splash, the severity of the injury and prognosis of the chemical damage on our body is determined by the following factors: 1. the toxic characters and concentration of the chemical; 2. the body surface size of the contacted area; 3. the duration of the chemical exposure; 4. the temperature of the chemical and environment; 5. if an appropriate decontamination has been exercised timely; 6. If needed, a proper medical treatment has been provided appropriately [11–13]. Lately, in industrial setting, upon chemical splash, immediate on site decontamination with appropriate rinsing solution to minimize the degree of the chemical burn and prevent further systemic toxicity has been considered as the principal purpose for emergent management [14]. To be able to achieve this purpose, at least, a rinsing solution to be used is supposedly need to hold the following capacities: 1. to be able to neutralize the pH (acid or alkali); 2. to be able to consolidate (or inactivate) the active compound; 3. to be able to wipe off the chemical left on the body surface [11–13].

In micro-electro-mechanical industries, TMAH is usually transported as a 25% solution (pH 13.5), which is diluted to a 2.38% solution at the point of use. TMAH easily dissociates, releasing hydroxide ions (OH^−^) and TMA^+^ [6].

TMA^+^ is a cholinergic agonist that binds to both nicotinic and muscarinic receptors in smooth muscle [15], skeletal muscle [16], cardiac muscle [17], and ganglionic cells [18], and this binding could result in acute cholinergic syndrome. The signs of acute cholinergic syndrome include bronchospasm, bronchorrhea, and bradycardia caused by muscarinic effects in the parasympathetic nervous system, respiratory muscle paralysis caused by nicotinic effects at the neuromuscular junction, and inhibition of the central respiratory center caused by nicotinic and muscarinic effects in the central nervous system [19]. However, TMA^+^ does not cross the blood-brain barrier [20]. An animal study found that pretreatment with mechanical ventilation could prevent death from a lethal dose of TMA^+^ [5], suggesting that acute respiratory failure is more likely the major cause of death following TMAH exposure.

It is difficult for TMA^+^ alone to penetrate through intact skin because of its polar nature and skin hydrophobicity. An animal study found that the 4-h lethal dose following dermal exposure was 85.9 mg/kg for 2.38% TMAH and 28.7 mg/kg for 25% TMAH [8]. A rat model revealed that dermal exposure to 2.75 M tetramethylammonium chloride (TMACl) alone was not fatal, whereas fatalities occurred following the dermal application of 2.75 M NaOH followed by 2.75 M TMACl [21]. This supports the theory that the intact skin serves as an effective barrier against the penetration of TMA^+^. However, a combination of TMA^+^ and a corrosive agent such as OH^−^ could cause superficial skin damage, allowing the penetration of TMA^+^ and eventually resulting in systemic toxicity.

Dermal absorption of TMA^+^ is influenced by the area and skin location, as well as the duration and concentration of exposure [21]. Systemic toxicity may develop in patients exposed to a relatively lower TMAH concentration (2.38% TMAH) [7, 22] or exposure involving a smaller TSBA (5% TBSA). Fatalities have been reported in patients exposed to 4% TMAH involving 7% TBSA [22], and 8.75% TMAH involving 12% TBSA [9] as well as 25% TMAH involving 7% TBSA [7]. Of frightened was that all the victims were announced of death upon their arrival to the emergency department, which was approximately within 30 min after the occurrence of TMAH splash.

TMAH has high water solubility; thus, emergent decontamination with copious amounts of tap water has been recommended, and this strategy is theoretically useful for limiting the extent of chemical skin injury and resultant systemic toxicity. However, water has only the *passive* decontamination effects of dilution and mechanical rinsing. In previously published reports, some patients who received nearly immediate irrigation with copious amounts of tap water still died [6, 7, 10].

Considering that the corrosive effects of OH^−^ may enhance the uptake of TMA^+^, as shown in case 1, using a flushing fluid that can *actively* bind and arrest the corrosive and toxic actions of both ions might be efficacious in limiting skin damage and thus tissue penetration. Diphoterine® solution is an amphoteric, slightly hypertonic, and chelating compound with multiple binding sites. It acts on acids and bases, as well as oxidizing and reducing agents (in addition to several other chemical substances), and it can prevent further injury to damaged tissues. It is nontoxic, it is not irritating to the skin or eyes, [14] and it does not release significant amounts of heat when reacting with various chemical agents [23]. An in vitro study revealed that to neutralize 1 mL of 25% TMAH to pH 9, the required amount of tap water was 17-fold greater than that for Diphoterine® solution [24]. In a reconstituted three-dimensional human skin model, cells were exposed to 25% TMAH for 30 s and then washed with either Diphoterine® solution or tap water. The results illustrated that 66.5% of the cells remained viable after Diphoterine® solution washing, versus 33.8% of cells after washing with tap water [24]. Diphoterine® solution has been shown to be more efficacious than water in decontaminating clinical eye/skin splashes with ammonium hydroxide and alkaline corrosives such as sodium and potassium hydroxides [24–27].

Currently, no antidote is available for TMAH poisoning. Atropine might be a potential antidote because it can reverse the muscarinic effects of acute cholinergic syndrome. In a prior study, rats were pretreated with 1 mg/kg atropine and then exposed to a lethal dose of TMAH. Survival time was prolonged, but atropine did not prevent mortality [5]. The reason why atropine did not prevent fatality may have been that the agent acted on muscarinic receptors but not on nicotinic receptors, and thus, respiratory muscle paralysis was not prevented. Another possibility was that the atropine dosage may have been insufficient to arrest the muscarinic effects of acute cholinergic syndrome. Thus, early administration of a sufficient dose of atropine might theoretically protect respiration in emergencies.

Upon dermal exposure to TMAH, respiratory muscle paralysis can develop rapidly without proper treatment, resulting in acute respiratory failure (i.e. less than 30 min after the exposure) [6, 7]. Our case 1, before the use of Diphoterine® solution, he had already developed significant systemic toxicity, even tap water wash has been carried out immediately on site. The merit of Diphoterine® use prevented him from progressively downward was witnessed by the victim himself and the co-worker who came along to assist him. The impressive response of Diphoterine® use observed in this case actually has encouraged us to approach the TMAH splash with Diphoterine® solution use for immediate on site decontamination in an active way. Very interestingly, in 2018, with its polyvalent property, Diphoterine® solution also had been listed and recommended in The Burns First Aid Guidelines, certified by the NHS of United Kingdom, as one of the first choice in managing the chemical burns. Aside from early decontamination to prevent or ameliorate the extent of systemic toxicity of TMAH, ensuring airway patency and supplying supplemental oxygen and mechanical ventilation as soon as possible may be life-saving in patients with TMAH poisoning. TMA^+^ has a short half-life of elimination [28], and the prognosis of severe TMAH poisoning should be favorable if patients are rapidly stabilized with supportive measures.

Patients with 25% TMAH exposure involving ≥5% TBSA developed significant and sometimes fatal systemic toxicity, patients with 25% TMAH exposure involving ≤1% TBSA did not develop systemic toxicity, and patients exposed to ≤25% TMAH who developed only first-degree skin injuries did not experience systemic toxicity. Whereas copious amounts of tap water have traditionally been used in frontline decontamination, our case 1 (with a 25% TMAH exposure of 5% TBSA and developed a significant systemic poisoning) has demonstrated that an amphoteric solution may be efficacious for skin decontamination on-site immediately to prevent or ameliorate such toxicity.

## Conclusions

TMAH acts as an alkaline corrosive and cholinergic agonist. Systemic signs attributable to TMA^+^ can rapidly lead to respiratory failure and death after dermal exposure. We had demonstrated that an amphoteric solution may be efficacious for skin decontamination on-site immediately to prevent or ameliorate such toxicity. This practice especially carries a valuable potential in managing victims (patients) who have been exposed to those chemicals with immediate life-threatening toxicity (e.g. TMAH), suggesting that its early utilization deserves further study.

### Limitations

This study had several limitations. First, this was a retrospective observational study, and the number of included cases was small. Second, most of the data were collected during telephone consultations; therefore, information might vary among the cases. Given the retrospective nature of the study design, incomplete data recording was possible. Not all cases of TMAH exposure in Taiwan are reported to PCC-Taiwan, which precluded an accurate assessment of the incidence and severity of TMAH poisoning.

## Data Availability

The raw data are patient contact records from the PCC-Taiwan. These are in a closed databank and not accessible to other than the staff of the PCC-Taiwan. The extracted datasets from this closed database which do not contain patient identifying information are available from the Corresponding Author (JFD) on reasonable request.

## Abbreviations

PCC-Taiwan: Taiwan Poison Control Center
TBSA: Total Body Surface Area
TMAH: Tetramethylammonium hydroxide
TMA^+^: Tetramethylammonium ion
TMACl: Tetramethylammonium chloride
